# Physicochemical and Mechanical Properties of Premixed Calcium Silicate and Resin Sealers

**DOI:** 10.3390/jfb14010009

**Published:** 2022-12-23

**Authors:** Naji Kharouf, Salvatore Sauro, Ammar Eid, Jihed Zghal, Hamdi Jmal, Anta Seck, Valentina Macaluso, Frédéric Addiego, Francesco Inchingolo, Christine Affolter-Zbaraszczuk, Florent Meyer, Youssef Haikel, Davide Mancino

**Affiliations:** 1 Department of Biomaterials and Bioengineering, INSERM UMR_S, Strasbourg University, 67000 Strasbourg, France; 2 Department of Endodontics, Faculty of Dental Medicine, Strasbourg University, 67000 Strasbourg, France; 3 Dental Biomaterials and Minimally Invasive Dentistry, Department of Dentistry, Cardenal Herrera-CEU University, CEU Universities, C/Santiago Ramón y Cajal, s/n., Alfara del Patriarca, 46115 Valencia, Spain; 4 Department Interdisciplinary of Bari, Università di Bari “Aldo Moro”, Giulio Cesare Square, 11, 70124 Bari, Italy; 5 Department of Endodontics, Faculty of Dental Medicine, Damascus University, Damascus 0100, Syria; 6 Laboratoire Energetique Mecanique Electromagnetisme, University of Paris Ouest, 50 Rue de Sèvres, 92410 Ville d’Avray, France; 7 ICube Laboratory, Mechanics Department, UMR 7357 CNRS, University of Strasbourg, 67000 Strasbourg, France; 8 Department of Conservative Dentistry and Endodontics, Department of Odontostomatology, Faculty of Medicine, Pharmacy and Odontology, Cheikh Anta Diop University, Dakar 10700, Senegal; 9 ESTA, School of Business and Technology, 90000 Belfort, France; 10 Luxembourg Institute of Science and Technology (LIST), Department Materials Research and Technology (MRT), ZAE Robert Steichen, 5 Rue Bommel, L-4940 Hautcharage, Luxembourg; 11 Pôle de Médecine et Chirurgie Bucco-Dentaire, Hôpital Civil, Hôpitaux Universitaire de Strasbourg, 67000 Strasbourg, France

**Keywords:** calcium silicate based sealer, resin sealer, mechanical properties, porosity

## Abstract

The aim of the present in vitro study was to evaluate specific mechanical and physicochemical properties of two calcium silicate based sealers, (AH Plus Bioceramic “AHPB”; Well-Root ST “WRST”), and a conventional resin sealer (AH Plus “AHP”). These aims were accomplished by assessing the porosity, pH, compression strength, roughness, wettability and cell attachment of the tested materials. The results were compared statistically using the one-way ANOVA test. Higher pH values were obtained in both AHPB and WRST compared to AHP at 3, 24 and 72 h (*p* < 0.05). A greater level of porosity and wettability was detected for both AHPB and WRST compared to the resin sealer AHP (*p* < 0.05). Evident cell growth characterized by elongated morphology was observed on the surface of AHPB and WRST, while only a thin layer of cells was seen on the surface of AHP. A significant lower compression strength and modulus were obtained in the specimens created using AHPB compared to those made with AHP and WRST (*p* < 0.05). The removal of calcium silicates may be quite tricky during endodontic retreatment. In conclusion, considering the limitations of the present in vitro study, both calcium silicate sealers demonstrated good physicochemical properties. However, the lower compression strength and modulus of AHPB may facilitate its removal and make the retreatment procedures considerably easier.

## 1. Introduction

Successful endodontic treatment consists of achieving an appropriate access cavity [1], good shaping [2], the use of proper irrigant solutions [3], and an optimal 3D obturation for root canal systems using gutta-percha and suitable sealers [4].

Several materials have been used as endodontic sealers to fill the canal and entomb the bacteria, so as to prevent re-infection of the root canal system [5]. These include epoxy resin, zinc oxide-eugenol, gutta-percha flow, and calcium silicate cements; such materials differ in terms of physicochemical, biological, and setting reactions [6]. Epoxy resin sealers have been considered as the gold standard for several years, and they are widely used in dental practice, as well as investigated to generate many scientific reports [7,8,9]. Calcium silicate (CS) materials, also defined as bioceramics, may set in moisture conditions, and they are often considered as a breakthrough in dental practice because they can be used in a wide range of endodontic treatments [9]. The advantageous antibacterial activity, biocompatibility, filling ability and physicochemical properties of CS materials make them the main choice in some specific surgical endodontics treatments [10]. Mineral trioxide aggregate (MTA) was the first bioceramic generation, which was introduced in the 1990s [11]. This cement is a mixture of Portland cement with bismuth oxide. The particle sizes of this cement (1.5–160 µm) were not able to supply an acceptable flow to adequately fabricate a pure bioceramic sealer. Thanks to nanotechnology, which was used to decrease the particle size, a new generation of bioceramic sealer was introduced in dental market [12]. CS sealers are usually used in powder–liquid format, so they require manual mixing; any alteration in this procedure could influence the physicochemical properties of the CS. Torres et al [13] showed that a change in the powder–liquid ratio of calcium silicate cement could increase the porosity and the solubility of this materials. Moreover, Cavenago et al [14] reported that a change in the powder–water ratio could influence the pH, setting time, calcium ion release, and radiopacity, and thus the physicochemical properties. Conversely, premixed CS sealers, which were available since 2007, do not need any manual mixing, so such a risk for modification of their properties due to improper mixing procedures is drastically reduced [15,16]. Moreover, these premixed sealers are ready to use and to be injected in the root canal space.

AH Plus (Dentsply Sirona, Ballaigues, Switzerland) is an epoxy resin based sealer available in a paste–paste form, which has been extensively investigated, both in in vivo and in vitro studies. [8,17]. Such a sealer has a short setting time (up to 8 h), proper film thickness, a greater dentin sealing ability than bioceramic materials and it could be used in warm obturation techniques during endodontic treatments [4,5,18,19]. AH Plus Bioceramic (Dentsply Sirona, Ballaigues, Switzerland) is a novel premixed bioceramic sealer, which has been introduced in the dental market. This sealer contains a reactive tricalcium silicate, but with a lower percentage of calcium silicate compared to existent bioceramic sealers due to the absence of di-calcium silicate within its composition [20]. Well-Root ST (Vericom, Chuncheon-si, Gangwon-Do, Korea) is a further modern premixed CS sealer, which contains calcium aluminosilicate as the reactive compound [21]. This injectable bioactive root canal sealer sets in moisture conditions in 25 min [22].

The physicochemical, mechanical, and biological characteristics of modern CS materials still require further investigation, especially for those that have been recently introduced in the market.

Therefore, the aim of the present in vitro study was to assess the cell attachement, compression modulus and ultimate strength, pH, porosity, roughness, and wettability of two novel calcium silicate based sealers compared to a conventional resin based sealer. The null hypothesis was that there would be no difference between the two modern CS sealers and the conventional resin based one, in terms of biological, mechanical, and physicochemical properties.

## 2. Materials and Methods

### 2.1. Materials

A conventional epoxy resin dental sealer currently available in the market, AH Plus (“AHP”, Dentsply Sirona, Ballaigues, Switzerland) and two novel pre-mixed calcium silicate based sealers, AH Plus Bioceramic (“AHPB”, Dentsply Sirona, Ballaigues, Switzerland) and Well-Root ST (“WRST”, Vericom, Chuncheon-si, Gangwon-Do, Korea) were used in the present study as per their manufacturers’ instructions (Table 1).

### 2.2. Specimen Preparations

**Figure 1:**
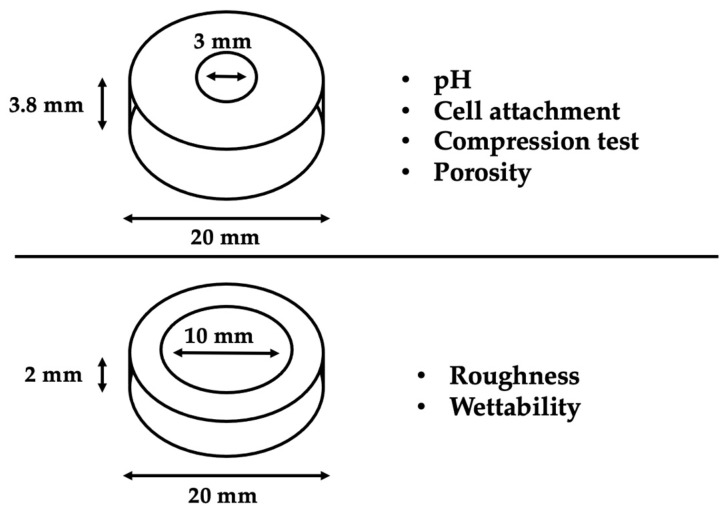
Schematical image shows the Teflon molds used to prepare the specimens for each test.

Teflon molds (height: 3.8 mm; diameter: 3 mm) were used to prepare the specimens for the following evaluations: pH, cell attachment and morphology, compression test, and porosity. Teflon molds (height: 2 mm; diameter: 10 mm) were used to prepare the specimens for the evaluation of wettability and roughness (Figure 1). The different sealers were injected directly using their injection tip into the different Teflon molds with glass slides underneath to provide a flat surface. All specimens were stored in the dark at 37 °C for 48 h to allow the materials to set properly.

### 2.3. Evaluation of pH

Each specimen was introduced into 10 mL vials containing distilled water and kept at 37 °C. A pH-meter (CyberScan pH 510, Thermo Scientific, Waltham, Massachusetts, USA), was used to measure the pH of media (n = 5) at 3, 24 and 72 h. The calibration of pH-meter was performed using standard solutions at pH 10, 4 and 7 (Hanna Instruments, Lingolsheim, France). Distilled water was used to rinse and eliminate the previous solution from the pH electrode [23].

### 2.4. Roughness and Wettability

The roughness of each surface was measured using a 3D digital profilometer (Keyence, Osaka, Japan) at 2500× magnification. The average roughness (Sa) (n = 5) was calculated using the Keyence 7000 VHX software (KEYENCE, Osaka, Japan) [24].

Further specimens (n = 3) were kept in dry conditions overnight and then submitted to the evaluation of the sorption time using a 5 µL drop of distilled water into the cement surface through a contact angle device (Biolin Scientific, Espoo, Finland). A horizontal camera was used to record the profile of the water drop and its absorption time, as previously described by Kharouf et al [25].

### 2.5. Cell Attachment and Morphology

Peridontal ligament stem cells (PDLs) were isolated from human alveolar ligament harvested from an extracted tooth by scrapping the middle 1/3 part of the root with a blade. Teeth were harvested from patient attending a private oral surgery practice based in Strasbourg, under the protocol CODECOH DC-2020-4345 approved by the French Ministry of higher education and research. Each patient was informed of the study’s procedure and objectives, and their non opposition to the use of their tooth was recorded in their chart by the oral surgeon. Alveolar ligaments were kept in complete medium + 0.5 mg/mL amphotericin B for transportation to the laboratory. PDLs were isolated following a standardized procedure: first alveolar ligaments are washed step with PBS, three times during 5 min, then digested for 2 h at 37 °C in an enzymatic solution containing dispase at 4 mg/mL and collagenase at 2.5 mg/mL, taking care to mix the solution manually every 15 min. One to four ligaments from the same patient are treated together in 50 mL of digestion solution. Once the ligament completely dissolved, the solution is filtered through a 70 µm cell sieve. The cells suspension is then centrifuged at 200 *g* for 5 min. Cells are resuspended in 5 mL of complete medium and incubated in 25 cm^2^ flask at 37 °C, 5 % CO_2_. Medium is changed every three days. Before use, cells were tested for CD90, CD73, CD34, CD45, HLA-DR, CD105, CD11b by flow cytometry to confirm their phenotype. Cells were cultivated in a specific medium (Gibco™ α-MEM, Thermo Fisher Scientific, Waltham, MA, USA) with 10% fetal bovine serum and 1% of penicillin–streptomycin (Dominique Dutscher, Bernolsheim, France). Activated media were prepared by incubating AHP, AHPB, and WRST specimens in 500 µL of complete medium for 8 days at 37 °C. The medium of the culture was changed every two days. After 8 days, the specimens were fixed as described in a previous study [26], by using a solution of 0.05 M glutaraldehyde in 4% cacodylate buffer for 2 h. Subsequently, the specimens were rinsed using a 4% cacodylate buffer three times, 5 min each, and subsequently dehydrated in a graded series of ethanol (35%, 50%, 70%, 95%, and 100%) for 3 min each. Finally, these were dried using the drying agent hexamethyldisilazane (HMDS). The specimens were transferred from 100% ethanol into a 1:1 solution of HMDS for 10 min, then transferred into 100% HMDS twice, 10 min each. All specimens were sputter-coated with gold–palladium (20/80) (20-nm thick layer) using a Hummer JR sputtering device (Technics, CA, USA). The specimens were observed analyzed at a magnification of 600× (working distance of 10 mm; high vacuum) [27] and images were taken for each sample for cell morphological characteristics through a scanning electron microscope “SEM” (Quanta 250 FEG scanning electron microscope “FEI Company, Eindhoven, The Netherlands”; 10 kV acceleration voltage of the electrons).

### 2.6. Porosity

The internal structures of AHP, AHPB, and WRST were inspected in 3D by means of micro-computed X-ray tomography (µCT) (EasyTom 160 from RX Solutions, Chavanod, France). The imaging procedures were executed at a voltage of 45 kV and a current of 160 mA, using a micro-focused tube equipped with a tungsten filament. The source-to-detector distance (SDD) and the source-to-object distance (SOD) were adjusted in such a way to obtain a voxel size of around 2.3 µm. Volume reconstruction was performed with the software Xact64 (RX Solutions) after applying geometrical corrections and ring artefact attenuation. Such an image process was performed using the Avizo software (ThermoFisher, Waltham, MA, USA) that allowed us to (i) de-noise the images with a median filter; (ii) segmentate the image intensity to reveal the objects of interest (here, the pores); (iii) remove insignificant small objects (below a size of 10 pixels from the segmented 3D data); and (iv) determine the 3D geometrical aspects of the objects of interest (volume and equivalent diameter) [28].

### 2.7. Compression Strength and Modulus

The specimens from the different groups (n = 8) were stored in water for 24 h and subsequently tested through the uni-axial compression test, in order to determine the stiffness of the cement and the maximum load before fracture. Such tests were performed using a universal electromechanical testing machine (Instron 3345, Norwood, MA, USA) device instrumented with a 1 kN cell force (Class 0.5 following ISO 7500-1), equipped with with a displacement sensor. The tests were performed at a constant crosshead speed of 0.5 mm/min [26]. The force in N and the crosshead displacement in mm were recorded during the test. The stress in MPa was calculated as force dived by initial section. The strain was obtained by dividing the crosshead displacement by the specimen initial length. The stress–strain curve was then plotted. The linear part of the stress–strain curve corresponds to the elastic behaviour. For each specimen, the compression modulus (Young’s modulus) is the slope of this linear part which is determined by a linear regression fitting. 

The compression strength was calculated in megapascals (MPa) according to the following formula:

*σc* = 4*P*/*πD*^2^ (1)

where *P* is the maximum recorded force during the test and *D* is the initial sample diameter.

### 2.8. Statistical Analysis

Statistical analysis was performed using the Minitab® 21 statical software- (Minitab, LLC, State College, PA, USA). The Shapiro–Wilk test was used to verify the normality of the data in all groups. However, when the normality was not verified as thus, an analysis of the variance on ranks, including a multiple comparison procedure (Tukey test), was used in order to determine whether significant differences existed in the compression modulus and strength values, water sorption tests, roughness measurements, and pH evaluations between the different composites. In all tests, a statistical significance level of α = 0.05 was adopted.

## 3. Results

### 3.1. pH Measurements

**Figure 2:**
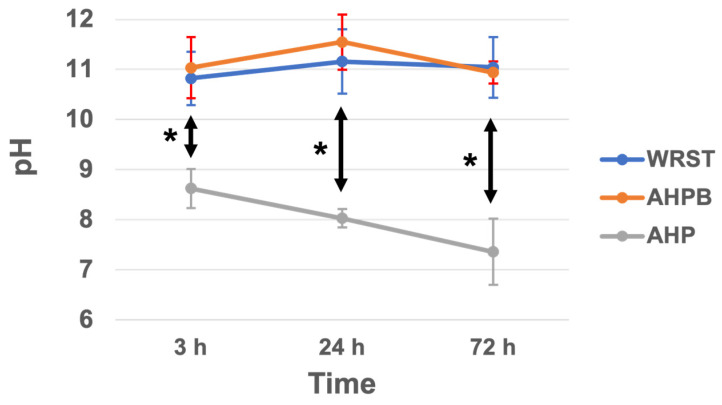
pH changes with time (3, 24 and 72 h) for water in contact with AH plus (AHP), AH Plus Bioceramic (AHPB) and Well-Root ST (WRST) at 37 °C. (* *p* < 0.05).

The pH of AHPB and WRST specimens immersed in 10 mL distilled water at 37 °C for 72 h presented no significant difference at any time period (3, 24 and 72 h) (*p* > 0.05). Conversely, significantly lower pH was observed for AHP compared to AHPB and WRST at 3, 24 and 72 h (*p* < 0.05) (Figure 2). 

### 3.2. Roughness and Wettability

**Figure 3:**
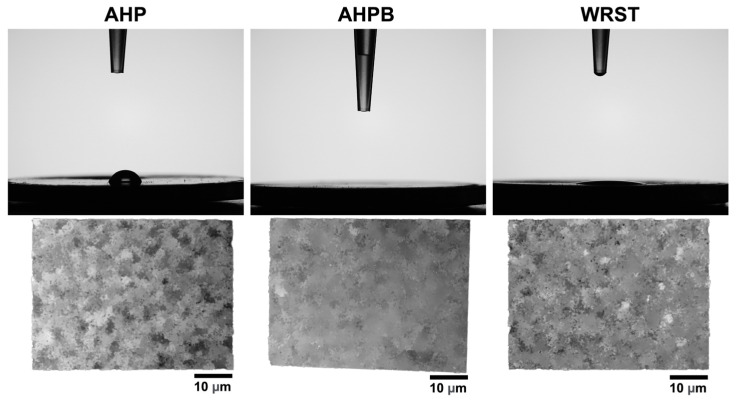
Water drop profile on AH plus (AHP), AH Plus Bioceramic (AHPB), and Well-Root ST (WRST) surfaces after 10 s. Digital micrographs of the different surfaces using KEYENCE 7000 VHX demonstrate the roughness of each material.

The profile of the 5 µL drop of distilled water was observed at 10 s. AHP had a less hydrophilic surface (64.9 ± 2.05)° compared to both CS sealers (*p* < 0.05). AHPB absorbed the drop of distilled water (0)° totally, whilst WRST presented a clear hydrophilic surface (10.4 ± 0.7)° (*p* < 0.05) (Figure 3, Table 2). AHP was characterized by a rougher surface than AHPB (*p* < 0.05). No statistical difference was found between both CS sealers (*p* > 0.05) (Figure 3, Table 2).

### 3.3. Cell Adhesion and Morphology

**Figure 4:**
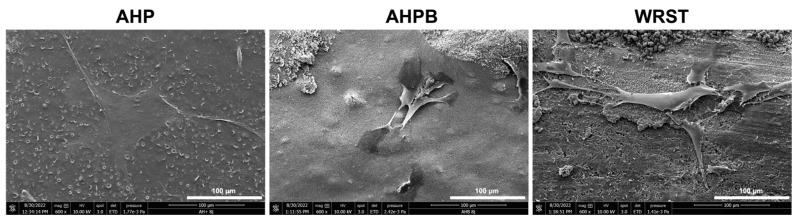
Morphology and cell adhesion on AH plus (AHP), AH Plus Bioceramic (AHPB) and Well-Root ST (WRST) surfaces after 8 days of culture.

Regarding the SEM qualitative analysis, more pronounced and thicker cells were observed onto the surface of AHPB and WRST, rather than that of AHP (Figure 4). Evident growth of spindle-like elongated cells were found on the surfaces of both CS tested materials.

### 3.4. Porosity

**Figure 5:**
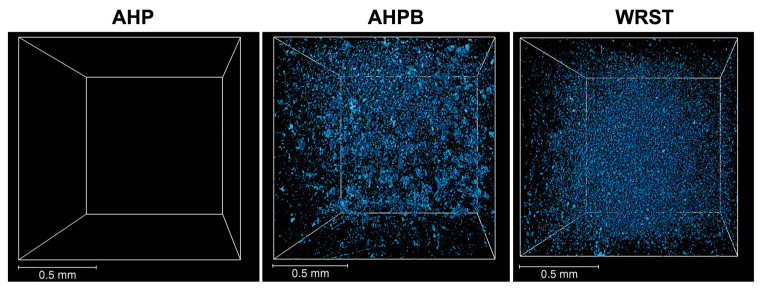
Volume rendering of the segmented pores (blue color) in: AH plus (AHP), AH Plus Bioceramic (AHPB) and Well-Root ST (WRST) obtained by X-ray tomography analysis. The scale bar corresponds to 0.5 mm in all images.

No voids were observed in AHP, while WRST demonstrated slightly higher porosity (1.00 %) compared to AHPB (0.68 %) (Figure 5, Table 3). Therefore, both calcium silicate based sealers were characterized by a greater porosity compared to AHP (resin based sealer).

**Figure 6:**
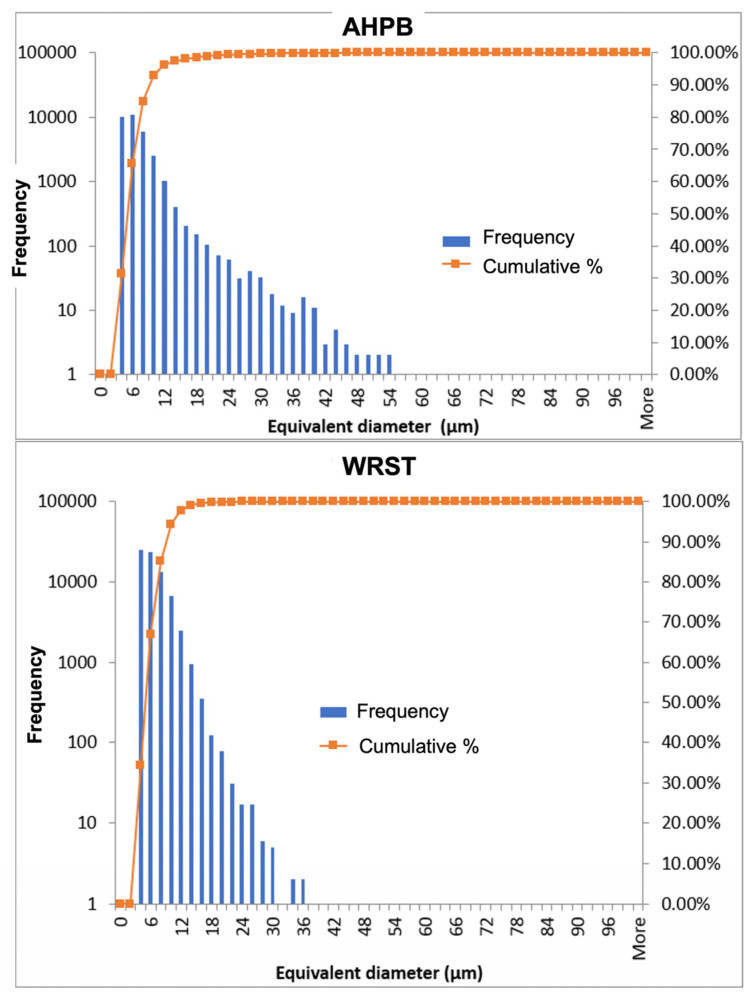
Equivalent pore diameter-Frequency curves obtained by X-ray tomography analysis in: AH Plus Bioceramic (AHPB) and Well-Root ST (WRST).

The equivalent diameter of the size of the pores has been calculated in both CS samples. Larger porosities (between 36 and 54 µm) were found for AHPB, while there was almost no porosity larger than 36 µm for WRST (Figure 6). Moreover, WRST had a higher frequency of small particles than AHPB.

### 3.5. Compression Strength and Modulus

**Figure 7:**
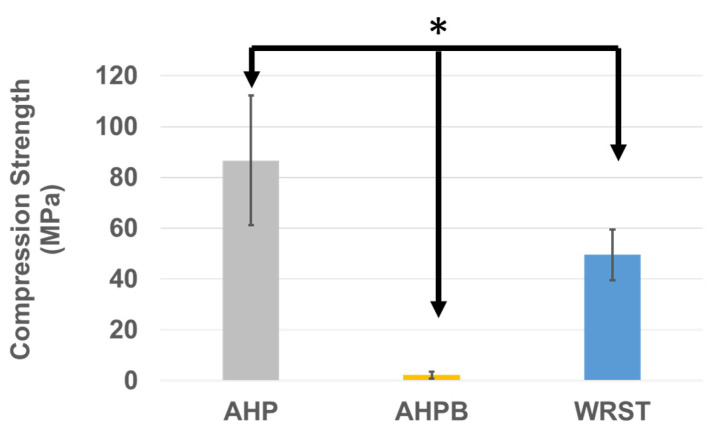
Compression strength values (means and standard deviations “MPa”) for AH plus (AHP), AH Plus Bioceramic (AHPB) and Well-Root ST (WRST) after 24 h of immersion in water at 37 °C. (* *p* < 0.05).

For compression strength (Figure 7), significant differences were found between the three materials. A significantly lower (*p* < 0.05) compression strength was obtained for AHPB compared to AHP and WRST after 24 h of immersion in water at 37 °C. AHP had higher compression strength values compared to WRST (*p* < 0.05). The lowest mean strength value was recorded for AHPB (2.22 ± 1.35 MPa).

**Figure 8:**
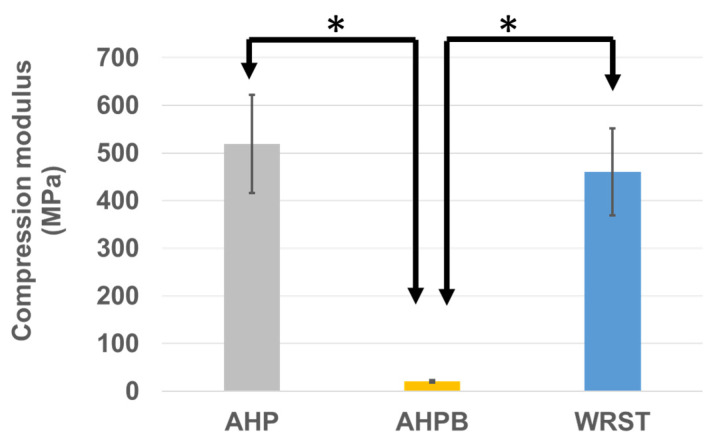
Compression modulus values (means and standard deviations “MPa”) for AH plus (AHP), AH Plus Bioceramic (AHPB) and Well-Root ST (WRST) after 24 h of immersion in water at 37 °C. (* *p* < 0.05).

For compression modulus (Figure 8), AHPB sealer presents a significantly lower modulus (20.72 ± 3.07 MPa) compared to AHP and WRST after 24 h of immersion in water at 37 °C (*p* < 0.05). However, no significat difference is obtained between the epoxy resin sealer APH (519.96 ± 102.98 MPa) and Well-Root ST sealer (460.49 ± 91.85 MPa).

## 4. Discussion

A recent study reported that approx. 51.7 % of the dentists around the world use calcium silicate cements for some specific endodontic treatments (e.g., pulp cupping, retrograde obturations) [29]. Currently, several calcium silicate based materials are available for endodontic clinical use [30], and recently, a novel CS sealer has been developed to replace the conventional AH Plus epoxy resin based sealer as an alternative to conventional CS sealers [6]. The conventional CS materials consist of high percentages of calcium silicate into their chemical composition, whilst the novel AHPB contains only 5–15% of tricalcium silicate. As mentioned in the introduction, this study aimed at assessing, in vitro, the biological, physicochemical, and mechanical properties of two novel CS sealers, and compare to those of a conventional epoxy resin based sealer; important characteristics and significant differences were found between the studied criteria of the tested materials. Thus, the null hypothesis postulated for this study that there would be no difference between the two modern CS sealers and the conventional resin based one must be rejected.

It was interesting to observe that both the modern CS sealers tested in this study (AHPB and WRST) had an alkaline pH at 3, 24 and 72 h, with no statistically significant difference (*p* > 0.05) between their values over time. In contrast, AHP demonstrated a significantly lower pH value (pH = 7–9) compared to AHPB and WRST (*p* < 0.05). The pH of water in contact with AHP was decreased after each measurement time (24 and 72 h). The pH values of AHP could be decreased by the fact that OH^-^ in the solution could be re-adsorbed at the surface of the cement. Therefore, in this case, a decrease in pH measurement could be observed. It is important to highlight that the alkaline pH may play an important role in terms of antibacterial activity and healing processes [16,24,25]. Accordingly, similar pH values for AHP and AHPD were reported in previous studies [8,20]. In the present study, WRST demonstrated slightly higher pH values than those reported by Yamauchi et al. [18]. Such a difference may be a consequence of the different method used in our study to evaluate the pH of the tested sealers. Any change in the quantity of media, the type of media (water, PBS), the shape and the dimensions of the specimens, the contact condition before and after setting time, the temperature, and the experimental design in replacing the storage solution in contact with the specimens could indeed influence the final results of the experiment [24]. However, it is important to state that an alkaline pH, along with the of Ca^2+^ released in the environment, may influence the bioactivity of CS materials. Therefore, further studies should be performed to evaluate the liberation of Ca^2+^ from the novel CS materials tested in this study, as well as their ability to induce precipitation of apatite-like crystals. The bioactivity of a material as defined previously [31], is a material which has been designed to induce specific biological activity, or a phenomenon by which a material modulates biological activity.

The absorption of a 5 µL drop of distilled water was measured on the surfaces of the tested AHP, AHPB, and WRST by means of a contact angle test; this was performed in order to evaluate the hydrophilicity of the tested materials. All specimens were stored in the dark at 37 °C for 48 h to allow the materials to set properly. After this period, the samples were put in the fume hood overnight in order to take out the remaining water from the sample surfaces. A cutoff on 90 degrees has been accepted to define the hydrophobicity (>90°) and hydrophilicity (<90°) of materials’ surfaces [32]. Both CS materials demonstrated a greater hydrophilic surface than AHP (epoxy resin). Moreover, the porosity was clearly revealed in these materials, whilst no evident pores were detected for AHP. Therefore, the higher contact angle of AHP than AHPB and WRST could be related to their chemical compositions and the difference in their porosity [25,33]. Indeed, the presence of resin in AHP can make this material more hydrophobic with fewer pores. Therefore, both CS sealers have better wettability and greater surface energy; this could influence their ability to adhere to dentin walls [33]. Our study is in accordance with the results published by Kapralos et al. [33], who showed similar contact angle values for AHP. Moreover, several studies performed on bioceramic materials demonstrated null or low contact angle values [24,25,33].

A significantly lower roughness was detected for AHPB compared to AHP and WRST (*p* < 0.05). This situation may be related to the percentage of filler, particles size, and chemical compositions of the tested materials. It is also important to state that greater roughness and wettability may play an important role in cellular attachment and proliferation [7,16,24]. Indeed, the SEM observations demonstrated that CS sealers presented several thicker and elongated cells on their surfaces compared to that of AHP, although this latter had the highest roughness, but the lowest wettability. Majhy et al [34] reported that higher roughness cannot be always considered as a beneficial factor for cellular adhesion, but the dimensions and the profile, as well as the composition of the rough surface, are more important. Therefore, CS sealers may favor cellular attachment and cell quality, and provide a superior biocompatibility.

**Figure 9:**
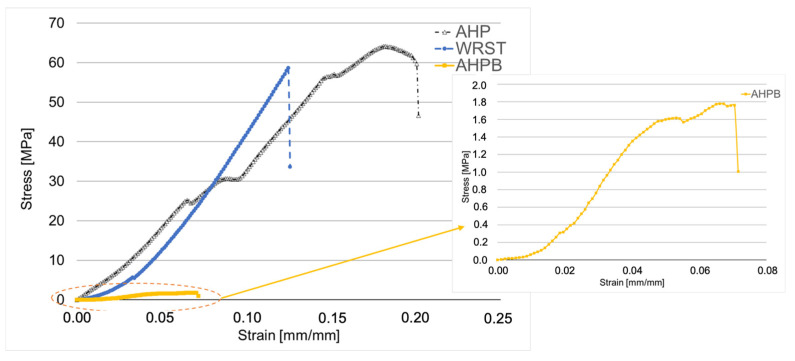
Stress–strain curve obtained by using the uni-axial compression test for AH plus (AHP), AH Plus Bioceramic (AHPB) and Well-Root ST (WRST) after 24 h of immersion in water.

Lowest compression modulus and strength were obtained when testing the specimens created using the AHPB sealer. In general, the lower the amount of calcium silicate in CS sealers, the greater the flexibility of sealer [35,36]. In addition, the most important phase in cement paste is the calcium silicate hydrate, which plays a role in the mechanical properties of the cements [35]. In accordance, Morejon-Alonso et al. [36] showed that the modification of apatite cements with tricalcium silicate might improve the mechanical properties and increase the bioactivity. Thus, the decrease in the mechanical properties for AHPB compared to WRST (both are CS sealers) may be explained by the fact that AHPB contains only tricalcium silicate in little quantity of 5–15 % [37] compared to conventional CS, which contain dicalcium silicate (7–15%) and tricalcium silicate (20–35%) [38]. Moreover, pore morphology, sizes, and porosity distribution, as well as connectivity and density percentages, could affect the compression strength of calcium silicate materials, as well as the chemical compositions [25]. AHP was characterized by the highest compression strength; this is mainly correlated to the presence of epoxy resin within its composition, which influenced its compression modulus. During the compression test, small pores generally deformed laterally and then they closed up. In the case of bigger and closer pores, the lateral deformation allows the pores to join, so resulting in progressive cracking and low stress failure. This behavior was commonly observed with the AHPB sealer (Figure 9).

The stress–strain curves (Figure 9) showed a brittle behavior for WRST sealer. The porosity percentage in WRST was the highest in comparison to AHP and AHPB. In addition, the pores within these materials were distributed in a manner to generate zones of stress concentrations and fragility in WRST. The presence of silicate of calcium increases the material stiffness of bioceramics. Significant differences in compression strength values (*p* < 0.05) were found between WRST (49.50 ± 10 MPa) and AHP (86.6 ± 25.5 MPa). However, in contrast to compression strength results, no significant difference for compression modulus was detected between AHP and WRST. The compression modulus of WRST becomes close to resin epoxy material (AHP). Thus, there was no significant difference between AHP and WRST in terms of compression modulus. The porosity does not contribute to the rigidity of the materials, but it was crucial in its overall toughness. No porosity was detected in the AHP resin compared to WRST, which presented the highest porosity density rate. This may explain the significant difference between WRST and AHP in terms of compressive fracture. In disagreement with a previous study that investigated the mechanical properties of two other CS sealers (Bioroot and Ceraseal), using the same experimental design of the present study [16], AHPB as CS based sealer, should have higher compression strength after immersion in water.

In clinical practice, the retreatment of calcium silicate materials is very difficult, especially in the last 2 mm of the root canal. The removal of such materials requires mechanical and chemical procedures [39]. Therefore, the “low” mechanical properties of AHPB as CS material could result in being more suitable in endodontic retreatments than when removing a conventional CS sealer. In contrast, the decreased mechanical properties of AHPB compared to WRST or AHP could play a negative role in reinforcing the instrumented root canal and the tooth, as well as the resistance of displacement of the cone during and after cone placement [40].

However, this is the first study to evaluate the mechanical properties of WRST and AHPB sealers. Further studies are required to evaluate the further chemical–physical characteristics of both sealers and the biological results of the reduction in CS quantity. Moreover, further research on the flowability and filling ability should be performed.

## 5. Conclusions

Within the limitations of the present in vitro study, both calcium silicate sealers demonstrated adequate physicochemical properties, and they could be considered as an appropriate choice in endodontic treatments. Higher pH alkaline and porosity, as well as hydrophily, were observed in both CS sealers (WRST and AHPB) compared to AHP. Moreover, lower mechanical properties were reported for AHPB compared to WRST and AHP. These decreased mechanical properties of AHPB would make retreatments considerably easier. Further studies should be performed to investigate the antibacterial activity, cytotoxicity, flowability, and filling ability of AHPB, which has lower tricalcium silicate percentages in its chemical composition.

## Figures and Tables

**Table 1:** Manufacturer, chemical composition and manipulation of the tested materials [6,18].

Sealer	Manufacturer	Lot	Manipulation	Chemical Composition
**AH Plus**	Dentsply Sirona, Ballaigues, Switzerland	2105000678	Auto mixed	Paste A: bisphenol-A epoxy resin, bisphenol-F, epoxy resin, calcium tungstate, zirconium oxide, silica, iron oxide pigments Paste B: dibenzyldiamine, aminoadamantane, tricyclodecane-diamine, calcium tungstate, zirconium oxide, silica, silicone oil
**AH Plus** **Bioceramic**	Dentsply Sirona, Ballaigues, Switzerland	KS211119	Premixed	Tricalcium silicate, lithium carbonate, zirconium oxide, dimethyl sulfoxide, thickening agents
**Well-Root ST**	Vericom, Gangwon-Do, Korea	WR180100	Premixed	Calcium aluminosilicate compound, zirconium oxide, filled and thickening agent

**Table 2:** Contact angles of 5 µL of water drop on AH plus (AHP), AH Plus Bioceramic (AHPB) and Well-Root ST (WRST) surfaces after 10 s. Mean and standard deviations of the roughness (Sa) of the tested materials. Superscript letters a, b, c and x, y indicate statistical significance (*p* < 0.05).

	AHP	AHPB	WRST	*p* < 0.05
Contact angle (°)	64.9 ± 2.05 ^a^	0 ^b^	10.4 ± 0.7 ^c^	a-b-c
Roughness (Sa)	1.08 ± 0.39 ^x^	0.38 ± 0.07 ^y^	0.58 ± 0.03	x-y

**Table 3:** Pore volume density of AH plus (AHP), AH Plus Bioceramic (AHPB), and Well-Root ST (WRST) calculated by X-ray tomography imaging.

Sealers	Pore Volume Density (%)
AHP	Non-detected
AHPB	0.68
WRST	1.00

## Data Availability

Not applicable.
